# Screening and identification of SipC-interacting proteins in *Salmonella* enteritidis using Gal4 yeast two-hybrid system in duck

**DOI:** 10.7717/peerj.7663

**Published:** 2019-09-13

**Authors:** Yu Zhang, Tiantian Gu, Yang Chen, Guoqiang Zhu, Wanwipa Vongsangnak, Qi Xu, Guohong Chen

**Affiliations:** 1 Joint International Research Laboratory of Agriculture & Agri-Product Safety of Ministry of Education, Yangzhou University, Yangzhou, China; 2 Jiangsu Key Laboratory of Zoonosis, Yangzhou University, Yangzhou, China; 3 Department of Zoology, Faculty of Science, Kasetsart University, Bangkok, Thailand

**Keywords:** Duck, PERP, TAB2, Salmonella enteritidis, SipC protein

## Abstract

The zoonotic pathogen *Salmonella* not only reduces the production performance in ducks, but also poses a serious threat to human health through eggs and pollutes water bodies through feces. SipC, an effector protein of type III secretion systems (T3SS) in *Salmonella*, mediates translocation of effectors into the eukaryotic host. However, the precise role of SipC effectors remains unknown in ducks. In this study, the SipC from duck granulosa cells (dGCs) was selected as bait, and the SipC-interacting proteins in *Salmonella* enteritidis (SE) were screened using Gal4 yeast two-hybrid system in duck. Twelve SipC-interacting proteins were identified. Among those, the p53-effector related to PMP-22 (PERP) and TGF-β activated kinase 1-binding protein 2 (TAB2) were selected to further confirm the function by GST pull-down *in vitro*. Over-expression of PERP resulted in not only increasing SE adhesion and invasion but also triggering the production of IL-1β and IFN-α in SE infected dGCs, while knock-down PERP showed the opposite tendency (*P* < 0.01). In addition, TAB2 significantly induced the production of IL-6, IL-1β, IFN-α, and INF-γ in SE infected dGCs (*P* < 0.05), but did not cause obvious changes in SE adhesion and invasion. When the * sipC* in SE was deleted, the activities of duck PERP and TAB2 were abolished because they could not bind to SipC. Taken together, although the protein of PERP and TAB2 can interact with SipC, their mechanisms were different in duck challenged by SE. Therefore, PERP was involved in SE invasion and inflammatory response of dGC ovaries, and TAB2 only contributed to dGCs inflammatory response, which provided critical insights about the mechanism in host- bacterium protein interactions during *Salmonella* invasion in duck.

## Introduction

*Salmonella* enteritidis (SE) is an important zoonotic pathogen that severely jeopardizes the success of livestock breeding and human health (Braden, 2006; Revolledo & Ferreira, 2012). Contaminated meat, including poultry, and eggs are the main vectors of human food-borne *Salmonella* outbreaks. China is a growing consumer of duck meat and eggs. As duck breeding increases, duck-borne bacterial diseases have become more common and complex each passing year. According to recent epidemiological reports, SE is the most frequent serotype isolated from ducks in developing countries (Cha et al., 2013; Gong et al., 2014). Transmission occurs through a vertical process from waterfowls, such as duck and geese, acting as long-term recessive carriers of SE. Infection not only affects egg production but also causes egg and water contamination, thereby endangering public health (Harker et al., 2014). Understanding mechanisms of SE invasion and persistent colonization of reproductive tissues of waterfowls is essential to develop strategies for reducing egg contamination, vertical transmission, and serious water pollution.

*Salmonella* harbor two specialized type III secretion systems (T3SS) that secrete effectors into the host cell to facilitate intracellular invasion and survival. Currently, the main type III secretion system believed to be involved in host cell invasion and systemic spread in poultry during SE infection is that encoded by pathogenicity island 1 (SPI-1 T3SS) (Hansen-Wester & Hensel, 2001). SipC, a major T3SS effector, mediates exocyst formation at sites of *Salmonella* invasion via interaction with multiple components on the plasma membrane. The level and timing of SipC expression dictate the consequences of SE infection and pathogenesis (Gong et al., 2010; Nichols & Casanova, 2010) .

SipC perturbs actin dynamics and effector translocation during *Salmonella* invasion of host cells (Hayward & Koronakis, 1999; Lara-Tejero & Galan, 2009; Myeni & Zhou, 2010). In addition, SipC directly binds to at least three components of the exocyst complex during *Salmonella* invasion, providing a “docking site” for exocyst formation and directing vesicle trafficking to the cell surface (Nichols & Casanova, 2010). For example, SipC regulates trafficking of a host membrane protein to the cell surface during *Salmonella* typhimurium (ST) infection (Hallstrom & McCormick, 2016). SipC is also a key regulator of the inflammatory response to *Salmonella* infection (Chang et al., 2007). Therefore, *Salmonella* evolved during long-term interactions with the host to form a variety of complex pathogenic mechanisms. However, little research exists on the mechanism of infection of *Salmonella* in ducks.

In this study, yeast two-hybrid technology with SipC as the bait protein was used to screen the host protein p53-effector related to PMP-22 (PERP) and TGF-*β* activated kinase 1-binding protein 2 (TAB2) as potential targets of the T3SS effector SipC. We verified SipC-PERP and SipC-TAB2 interactions by GST pull-down assays. The co-regulatory factors PERP and TAB2 were further investigated for their role in promoting SE invasion and inflammatory response of duck granulosa cells (dGCs). Our results demonstrated interactions between effector and host cell proteins, offering novel evidence of transovarian transmission between SE and host interaction.

## Materials and Methods

### Ethical statement

All animal experiments used in this study were approved by the Institutional Animal Care and Use Committee of Yangzhou University (Jiangsu, China) and were strictly implemented according to the regulations for experimental animals. An ordinary housing facility was used and was consistent with the national standard, Laboratory Animal Requirements of Environment and Housing Facilities (GB 14925-2001). Laboratory animal care and the animal experiment protocols and conditions conformed to the Jiangsu Administration Rule for Laboratory Animal Use.

### Isolation and culture of dGCs

Healthy, *Salmonella*-free, 26-week-old Shaoxing ducks were collected from the National Waterfowl Conservation Farm (Taizhou, Jiangsu, China). Separation and cultivation of dGCs was performed as previously described (Gilbert et al., 1977). Briefly, 10 to 15 adult prehierarchical follicles (small yellow follicles), which have thicker granulosa cell layer (Yang et al., 2019) of egg-laying ducks, were collected under aseptic conditions and rinsed with Ca^2+^- and Mg^2+^-free phosphate-buffered saline (PBS) to remove the yolks and vitelline membrane as fully as possible. Thereafter, the tissue was cut into 1–2 mm^3^-sized blocks, digested with 1 mg/ml collagenase (Type II; Sigma Chemical Company, St. Louis, MO, USA) at 37 °C for 5 min, and filtration through a 200-µm nylon filter. Filtered suspensions were centrifuged twice at 67 × g for 5 min. Pellets were washed with M199 media to remove the remaining collagenase and cell debris, resuspended in three mL 50% Percoll, and centrifuged at 421 × g for 15 min, after which the cell layer was aspirated. Granulosa cell suspensions were prepared by adding a pre-configured M199 media (5% fetal calf serum, two mmol/L L-glutamine, five µg/mL transferrin, 10 µg/mL insulin, 1.75 mM HEPES) and counted following staining with 0.1% trypan blue. Suspensions with a cell survival rate greater than 90% were used for experiments. Cells were used for follow-up experiments in disposable culture flasks after 24 h when the completely adherent. Cell purity was initially determined by hematoxylin and eosin (H&E) staining.

### Construction of yeast two-hybrid cDNA library of dGCs

cDNA and yeast two-hybrid libraries were constructed in collaboration with Shanghai OE Biotech. Total RNA was extracted from the dGCs using TRIzol (Invitrogen, Carlsbad, CA, USA), and mRNA was isolated using an Oligotex mRNA Midi Kit (Invitrogen). RNA integrity and size distribution range of mRNA were detected by 10 g/L denaturing gel electrophoresis. Total RNA and mRNA concentrations were measured using a Thermo Nanodrop nucleic acid analyzer.

In order to construct a cDNA library containing three reading frames, the corresponding attB linker sequences at the 5′and 3′ends of the corresponding cDNA to ensure that the cDNA was translated in the correct reading frame (Table 1). The CloneMiner II cDNA Library Construction Kit (Invitrogen) was used according to the manufacturer’s instructions. Column chromatography was performed to fractionate and collect cDNA. Collected cDNA fragments were digested using a pDONR 222 and BP Clonase II enzyme mix for BP recombination. Recombination products were then transformed into *E. coli* DH10B competent cells by electroporation and cultured at 37 °C and 4 × g for 1 h, and primary library bacteria were obtained. The insert size and recombination rates of the positive clones were determined by PCR.

**Table 1:** PCR primer sequences. To construct a cDNA library containing three reading frames, purified mRNAs, biotin-attB2-Oligo(dT) primer, 5 × first-strand buffer, DTT, dNTPs, and SuperScript III reverse transcriptase were added to synthesize the first strand of cDNA. After that, a second strand of cDNA was synthesized by adding 5 × second-strand buffer, *Escherichia coli* (*E. coli*) DNA ligase, *E. coli* DNA polymerase I, and *E. coli* RNaseH to the first-strand synthesis reaction solution. Additional reagents, including 5 × adapter buffer, attB1 adapter, DTT, and T4 DNA ligase were added to connect the attB1 recombination linker.

Primer	Sequence (5′–3′)	Usage
*attB*1 (RFα)	TCGTCGGGGACAACTTTGTACAAAAAAGTTGG	Adapters
*attB*1 (RFβ)	TCGTCGGGGACAACTTTGTACAAAAAAGTTGGA	
*attB*1 (RFγ)	TCGTCGGGGACAACTTTGTACAAAAAAGTTGGAA	
*attB*2	ACCCAGCTTTCTTGTACAAAGTGGT	
M13 F (−20)	GTAAAACGACGGCCAG	Primary library identification
M13 R	CAGGAAACAGCTATGAC	
pGADT7-F (T7)	TAATACGACTCACTATAGGGCGAGCGCCGCCATG	Expression library identification
pGADT7-R (ADR)	GTGAACTTGCGGGGTTTTTCAGTATCTACGATT	
*sipC*-F	CGCCATATGATGTTAATTAGTAATGTGGGA	Bait gene
*sipC*-R	TGCACTGCAGTTAAGCGCGAATATTGCCT	
*sipC*-F	CGCGGATCCATGTTAATTAGTAATGTGGGA	Fusion plasmid
*sipC*-R	CCGGAATTC TTAAGCGCGAATATTGCCT	
*perp*-F	CGCGGATCCATGGTGGTGTGCAGCCTCGC	Fusion plasmid
*perp*-R	CCGGAATTCTCAATTTAAGTATGGTGCTCTCT	
*tab2*-F	CTAGCTAGCATGGCCCAAGGAAGCCAGCAA	Fusion plasmid
*tab2*-R	TGCACTGCAGGAAATGCCTGGGCATTTCAC	
*IL-6*-F	AAAGCATCTGGCAACGAC	RT-qPCR
*IL-6*-F	GAGGAGGGATTTCTGGGT	
*IL-1*β-F	CCGAGGAGCAGGGACTTT	RT-qPCR
*IL-1*β-R	AGGACTGTGAGCGGGTGTAG	
*TNF-*α-F	GATGGGAAGGGGATGAAC	RT-qPCR
*TNF-*α-R	ACTGAGCCAGATTGTTACCC	
*IFN-*γ-F	CCTTTACCAAGAACAACCTG	RT-qPCR
*IFN-*γ-R	GCCTTGCGTTGGATTTTC	
*GAPDH-F*	TGCTAAGCGTGTCATCATCT	RT-qPCR
*GAPDH-R*	AGTGGTCATAAGACCCTCCA	

For construction of a yeast two-hybrid cDNA expression library, the primary library containing 5 × 10^6^ to 1 × 10^7^ positive clones was inoculated into 100 mL broth containing kanamycin (final concentration 50 µg/mL) at 30 °C and 4 × g until OD_600_ reached 1.0. The primary library plasmids were extracted using a PureLink HQ Mini Plasmid DNA Purification Kit (Invitrogen), and subsequently diluted to 300 ng/µL, and then added one µL to compatible vectors pGADT7-DEST, LR Clonase II Mix, and ddH_2_O to carry out the LR recombination reaction. The recombination products were transformed into *E. coli* DH10B competent cells by electroporation and cultured at 37 °C and 4 × g for 1 h to obtain bacteria expressing the library. The size of the inserts and recombination rates of the positive clone were determined by PCR.

### Construction of the bait plasmid pGBKT7-*sipC* and detection of its self-activation activity

According to the sequence of SE *sipC* (Genbank ID: NC_003197.2), specific primers were synthesized with the upstream primer harboring an *Nde* I restriction site, and the downstream primer harboring a *Pst* I restriction site and a stop codon. The recombinant pGBKT7-*sipC* plasmid (bait plasmid) was confirmed by double restriction enzyme digestion and sequencing (Sangon Biotech, Shanghai, China). The recombinant pGBKT7-*sipC* plasmid was transformed into yeast strain Y2HGold as per the manufacturer protocol of the Yeastmaker™ Library Construction & Screening Kit (Clontech, Mountain View, CA, USA). Transformants were screened on SD/-Trp/X-*α*-Gal agar plates at 30 °C for 3–5 days. Growth, size, and color changes of colonies were assessed to determine if the bait plasmid had self-activation activity. Positive (pGBKT7-53/pGADT7-T) and negative (pGBKT7-Lam/pGADT7-T) controls were coated on SD/-Trp/-Leu/X-*α*-Gal media plates, respectively. Only bait plasmids without auto-activation can be used in yeast two-hybrid screening.

### Yeast two-hybrid screen based on co-transformation of bait and prey plasmids

In order to screen host proteins that interact with the SipC bait in the yeast two-hybrid system, a cDNA library of dGCs, pGBKT7-*sipC*, and the library plasmid were co-transformed into Y2HGold using a Yeastmaker™ Library Construction & Screening Kit according to the manufacturer protocol. Co-transformants were then grown on higher-stringency SD/-Ade/-His/-Leu/-Trp/X-*α*-Gal/ABA agar plates at 30 °C for 3–5 days. Single blue colonies were selected and serially passaged three times on SD/-Ade-His-Leu-Trp/X-*α*-Gal/ABA plates. Positive colonies were thereafter re-seeded three times in SD/-Ade-His-Leu-Trp liquid media. Prey plasmids were extracted from putatively positive clones using the Easy Yeast Plasmid Isolation Kit (Clontech).

### Confirmation of the positive clones of interactions

To confirm interactions, co-transformations of pGBKT7-*sipC* bait into Y2HGold with each prey plasmid in putatively positive hits were performed. Briefly, the prey plasmids were extracted from putatively positive clones using an Easy Yeast Plasmid Isolation Kit (Clontech, Mountain View, CA, USA). Subsequently, each prey plasmid was transformed into *E. coli* DH5α competent cells (Transgen, Beijing, China) and purified from transformants growing on selected LB/ampicillin agar plates using a Plasmid Mini Kit I (Omega Bio-Tek, Norcross, GA, USA). Each putatively positive prey plasmid was co-transformed with pGBKT7-*sipC* bait and pGBKT7 plasmids into Y2HGold. Cotransformants were grown on SD/-Ade-His-Leu-Trp/X-*α*-Gal/ABA plates to test for interactions. The cotransformant containing pGADT7-T and pGBKT7-Lam grown on an SD/-Ade-His-Leu-Trp/X-*α*-Gal/ABA plate was used as a negative control, and the cotransformant containing pGADT7-T and pGBKT7-53 grown on SD/-Ade-His-Leu-Trp/X-*α*-Gal/ABA was used as a positive control. True positive interactions were indicated by blue colonies under these conditions. Positive prey plasmids were sequenced, and results were blasted against NCBI databases.

### GST pull-down assay

GST pull-down assays were performed as previously described (Hallstrom et al., 2015). Briefly, GST-SipC fusion plasmid (pGEX-6P-GST containing the GST-tagged C-terminus with SipC) was transformed to BL21 (DE3) followed by cultured induction with IPTG to express the GST-SipC fusion protein. Then, the recombinant bacteria BL21 were centrifuged at 4,315 × g. The pellet was resuspended in lysis buffer (100 mM NaCl, 25 mM Tris, 1% TritonX-100, 0.2 mM lysosome, 1 mM PMSF, 10% glycerin, pH 8.0), sonicated five times (1.5 w/s) and centrifuged at 9,710 × g at 4 °C for 10 min. Pre-cleared supernatants were incubated with glutathione-sepharose affinity matrix beads for 2 h at room temperature. Plasmid constructs pEGFP-PERP and pEGFP-TAB2 were transfected into dGCs using Lipofectamine 2000. The transfected dGCs were lysed and incubated with the SipC-GST-bound beads overnight at 4 °C with end-over-end rotation according to the manufacturer instructions. After washing steps with 1 × PBS, GST-SipC protein complexes were eluted with 10 mM reduced glutathione which was dissolved in lysis buffer. The eluates were then diluted in 20 µL 5 × SDS protein-loading buffer (125 mM Tris-HCl, pH 6.8, 4% w/v SDS, 20% glycerol, 100 mM DTT, 0.02% w/v bromophenolblue) boiling for 5 min. SDS-PAGE detection and Western bolting analysis were then performed. PERP polyclonal antibody (1:2,000; Abcam, Cambridge, MA, USA) and TAB2 polyclonal antibody (1:1,000; Abcam, Cambridge, MA, USA) were used as primary antibodies, and goat anti-rabbit IgG-HRP (1:5,000) was used as secondary antibody. Diaminobenzidine (DAB) reaction was used for color development.

### Transient transfection

PERP and TAB2 cDNA were amplified by RT-PCR using gene-specific primers (Table 1). The PCR products were cloned into the expression vector pEGFP-N1. A small interfering RNAs (siRNA) targeting PERP (pGPU6/GFP/Neo-PERP) and TAB2 (pGPU6/GFP/Neo-TAB2) (GenePharma, Shanghai, China) were used to decrease PERP and TAB2 expression, respectively. The interference efficiency of the three targeted interference vectors was detected by qPCR, and the most effective siRNA was used for subsequent experiments. One of the three PERP-siRNAs, TAB2-siRNAs or the negative control siRNA were transfected into dGCs using Lipofectamine 2000 (Invitrogen, Grand Island, NY, USA) according to manufacturer instructions, respectively.

### Quantitative real-time PCR analysis

Primers for qRT-PCR specific to duck immune response genes *IL-6*, *IL-1β*, *TNF*-*α*, and *IFN*-*γ* are listed in Table 1. The housekeeping genes glyceraldehyde-3-phosphate dehydrogenase (*GAPDH*) was used as reference controls to normalize the expression level. Total RNA was extracted using TRIzol Reagent (Takara, Dalian, China), according to the manufacturer’s instructions. We performed qPCR using an ABI 7500 system (Applied Biosystems, Foster City, CA, USA). Each reaction volume contained 10 µL 2 × SYBR Premix Ex Taq II (Takara), 0.4 µL forward primer (10 µM), 0.4 µL reverse primer (10 µM), 0.4 µL 50 × ROX Reference Dye II (Takara), 2 µL cDNA, and ddH_2_O to 20 µL. The amplification program was performed as follows: initial denaturation at 95 °C for 30 s, followed by 40 cycles of denaturation at 95 °C for 5 s and annealing at 60 °C for 34 s. To analyze the specificity of the amplified products, we collected multiple information points for melting curve analysis. The program was as follows: 95 °C for 15 s, 60 °C for 1 min, 95 °C for 15 s, and 60 °C for 15 s. Data were analyzed by the 2-^ΔΔCt^ relative quantitative method.

### Adhesion and invasion assays

The adhesion assay was performed as previously described (Jouve et al., 1997; Lane & Mobley, 2007). Briefly, bacteria were grown to an OD_600_ nm of 2.0 in LB broth at 37 °C. dGCs were seeded in 96-well tissue culture plates at a concentration of 1 × 10^5^ cells per well and grown for 24 h. Cell monolayers were washed three times with sterile PBS (pH 7.2). Then, dGCs were incubated with 100 µL bacterial suspension in DMEM with multiplicity of infection (MOI) of 10 per well of a 96-well plate at 37 °C. Infections were performed in triplicate. After 1 h incubation, the infected cell monolayer was gently washed three times with PBS to remove loosely adherent bacteria. For invasion assays, PBS contained 50 µg/mL gentamicin was used to kill noninvasive bacteria. After 1 h, cells were lysed with 0.5% Triton X-100 (Solarbio, Beijing, China) for 30 min. Lysates were serially diluted and plated on LB-agar plates for bacterial growth. The assay was performed in triplicate.

### Statistical analysis

Each experiment was repeated at least three times and the data were analyzed using an independent-sample *t* test (SPSS version 13).

## Results

### Construction of yeast two-hybrid cDNA library of dGCs

The dGCs were in round or oval shapes at first, after 24 h in culture, the monolayers adhered to the wall and spread out like pebbles. Granular material was observed in the center of cells. Small particles were observed by H&E staining (Fig. S1). Purified dGCs (2 × 10^7^) were used for cDNA library construction. A diluted primary library solution (50 µL at 1:1000 dilutions) was spread on kanamycin-resistant LB plates for 14 h. According to the formula, the titer of the primary library was calculated to be 2. 97 × 10^6^CFU/mL, and the total library capacity was 1. 19 × 10^7^CFU. Twenty-four clones were randomly picked for PCR analysis. All clones were positive, with different sizes of fragments inserted. The recombination rate of the primary library was 100%, and the average size of insert was >one kb (Fig. S2A). The diluted expression library (50 µL at 1:1,000) was plated on ampicillin-containing LB plates for 14 h. According to the formula, the expression library titer was 1.36 × 10^6^ CFU/mL, and the total library capacity was 5.44 × 10^6^ CFU. Twenty-four clones were randomly selected for PCR analysis. All selected clones were positive, with different sizes of fragments inserted, indicating that the recombination rate of the expression library was 100%. The average size of insert was >one kb (Fig. S2B). These results indicated that the cDNA library could be used for yeast two-hybrid screening.

### Screening of *Salmonella* enteritidis SipC-interacting proteins using a Gal4 yeast two-hybrid system

**Figure 1:**
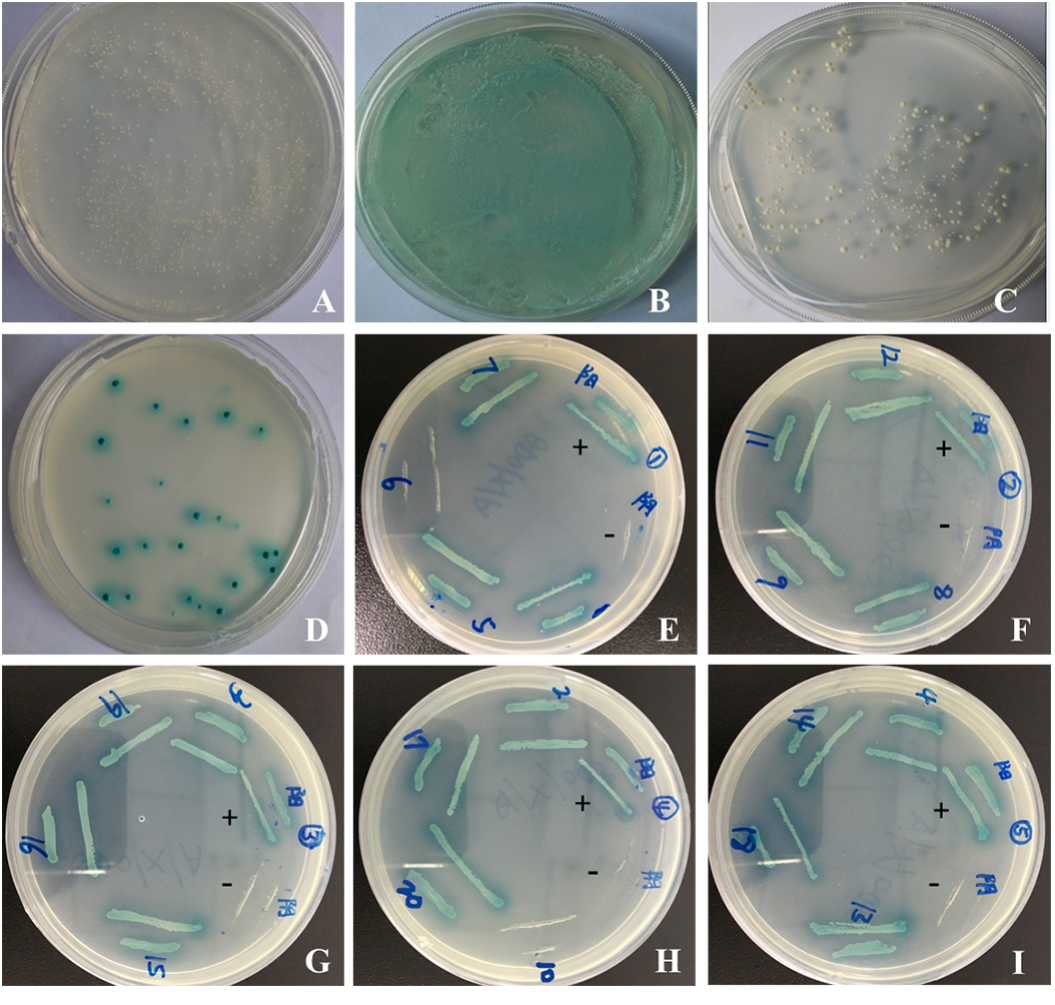
Yeast two-hybrid screening and confirmation of the interaction. (A–C) Detection of self-activation activity of bait plasmid pGBKT7-*sipC*. (A) negative control plasmid (pGBKT7-Lam/pGADT7-T) chromogenic experimental results; (B) positive control plasmid (pGBKT7-53/pGADT7-T) chromogenic experimental results; (C) plasmid pGBKT7-*sipC* chromogenic experimental results. (D) cotransformants screened; (E–I) one-to-one interaction verification.

pGBKT7-*sipC* demonstrated successful insertion of the gene into the yeast expression vector pGBKT7 by sequencing. To test the auto-activation activity of the bait protein in yeast cells, the pGBKT7 and pGBKT7-*sipC* bait plasmids were transformed into Y2HGold and subsequently the transformants were grown on SD/-Trp-Leu/X-*α*-Gal media plates. As a negative control, the empty vector failed to activate the reporter gene (Fig. 1A). Transformants containing the pGBKT7-53 and pGADT7-T control vectors were grown on SD/-Leu-Trp/X-*α*-Gal media and were blue in color as a positive control experiment (Fig. 1B). Although transformants containing pGBKT7-*sipC* grew on SD/-Trp/X- α-Gal media, not blue colonies were observed. The bait gene did not activate the reporter genes Ade2 and His, indicating an absence of self-activation in pGBKT7-*sipC* (Fig. 1C).

After cotransformation of pGBKT7-*sipC* and prey plasmids and growth on SD/-Ade-His-Leu-Trp/X-*α*-Gal/ABA plates, 20 blue clones were obtained. Subsequently, these 20 prey plasmids were isolated from their corresponding colonies and rescued through transformation of *E. coli* DH5 α cells. To eliminate false positive hits, each of the 20 prey plasmids was co-transformed with pGBKT7-*sipC* into Y2HGold cells. Cotransformants were cultivated on SD/-Ade-His-Leu-Trp/X-α-Gal/ABA plates. Each of the 20 cotransformants grew as blue colonies on SD/-Ade-His-Leu-Trp/X-*α*-Gal/ABA plates. Meanwhile, each of the 20 prey plasmids was co-transformed with pGBKT7 plasmid into Y2HGold cells, no colony growth was observed on SD/-Ade-His-Leu-Trp/X-*α*-Gal/ABA plates. Based on the above results, 12 host proteins were identified to interact with SipC (Figs. 1D–1I). To confirm the nucleotide sequence information of the identified host proteins interacting with SipC, the 20 prey plasmids were sequenced and analyzed using the BLAST tool in NCBI (DOI 10.6084/m9.figshare.7844426.v1). Twelve of the interaction plasmids were identical to SipC (Table 2).

**Table 2:** Analysis of positive gDNA cDNA library of pGBKT7-*sipC* and *Anas platyrhynchos* dGCs cDNA library.

Gene name	Gene bank	ORF true or not	Pairwise
RPA2	XM_005025276.2	TRUE	Blue
PERP	XM_005017738	TRUE	Blue
ANAPC11	XM_011600127.1	TRUE	Blue
RYBP	XM_005025691.2	TRUE	Blue
Peripheral-type benzodiazepine receptor-associated protein 1	XM_005028332.2	Not	Blue
MYH10	XM_005030657.2	TRUE	Blue
RNA-binding	XM_013107009.1	TRUE	Blue
DNAJC7	XM_005027190.2	TRUE	Blue
tRNA wybutosine-synthesizing	EOB07987.1	TRUE	Blue
CCNDBP1	XM_013105152.1	TRUE	Blue
TAB2	XM_005008888.2	TRUE	Blue
BET1	XM_005010962.2	Not	Not blue
MCM6	XP_012955805.1	TRUE	Blue
CEP131	XM_013098518.1	TRUE	Not blue
MZT1	XM_013098512.1	TRUE	Blue

**Notes.**

“not” and “not blue” mean that there was no interaction relationship between the two proteins.

### Identification of *Salmonella* enteritidis SipC-interacting proteins PERP/TAB2 in using GST pull-down assays

**Figure 2:**
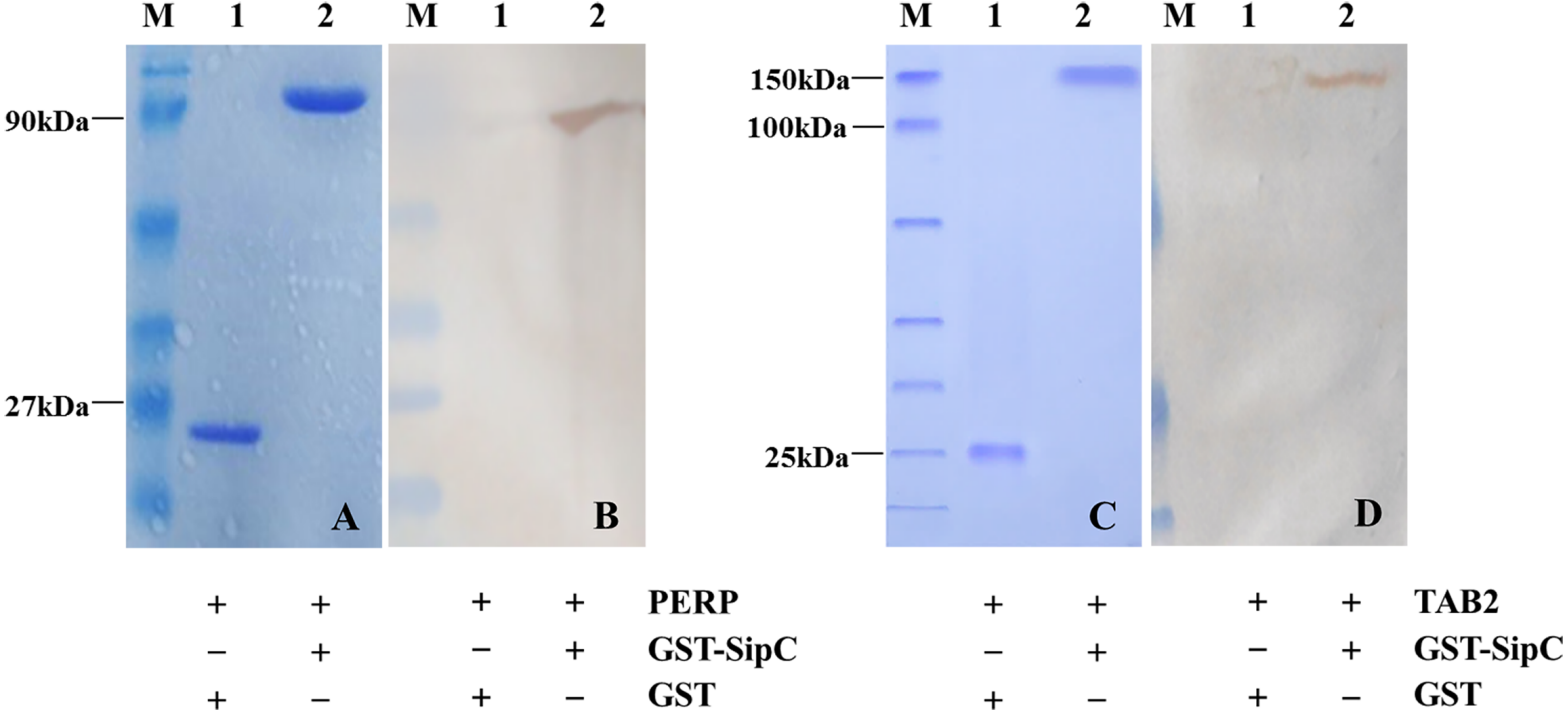
SipC interacts with PERP and TAB2 by GST-pull down. (A–B) SipC interacts with PERP, (A) Coomassie blue staining of GST (line 1), recombinant prokaryotic-expressed GST-SipC and eukaryotic-expressed PERP protein lysed from dGCs transfected with pEGFP-PERP (line 2); (B) immunoblotting with anti-PERP antibody by DAB staining, GST (line 1), recombinant prokaryotic-expressed GST-SipC and eukaryotic-expressed PERP protein lysed from dGCs transfected with pEGFP-PERP (line 2). (C–D) SipC interacts with TAB2, (C) Coomassie blue staining of GST (line 1), recombinant prokaryotic-expressed GST-SipC and eukaryotic-expressed TAB2 protein lysed from dGCs transfected with pEGFP-TAB2 (line 2); (D) immunoblotting with anti-TAB2 antibody by DAB staining, GST (line 1), recombinant prokaryotic-expressed GST-SipC and eukaryotic-expressed TAB2 protein lysed from dGCs transfected with pEGFP-TAB2 (line 2).

Detection of protein–protein interaction using the GST fusion protein pull-down technique. Full length SE SipC cDNA was introduced into a GST expression vector, downstream of the glutathione-S-transferase coding part. Following expression in bacteria, the corresponding fusion protein and the transfected cell lysates were prepared, then their purity and interaction relationship were detected by SDS-polyacrylamide gel and Western bolting analysis. GST pull-down assays revealed that GST did not bind to host protein PERP/TAB2. However, GST-SipC bound specifically to PERP/TAB2 with normal expression of each protein. These results support the direct and specific interaction between the SE effector protein SipC and the host protein PERP/TAB2 (Fig. 2).

### PERP interacts with SipC to promote SE adhesion and invasion to dGCs

**Figure 3:**
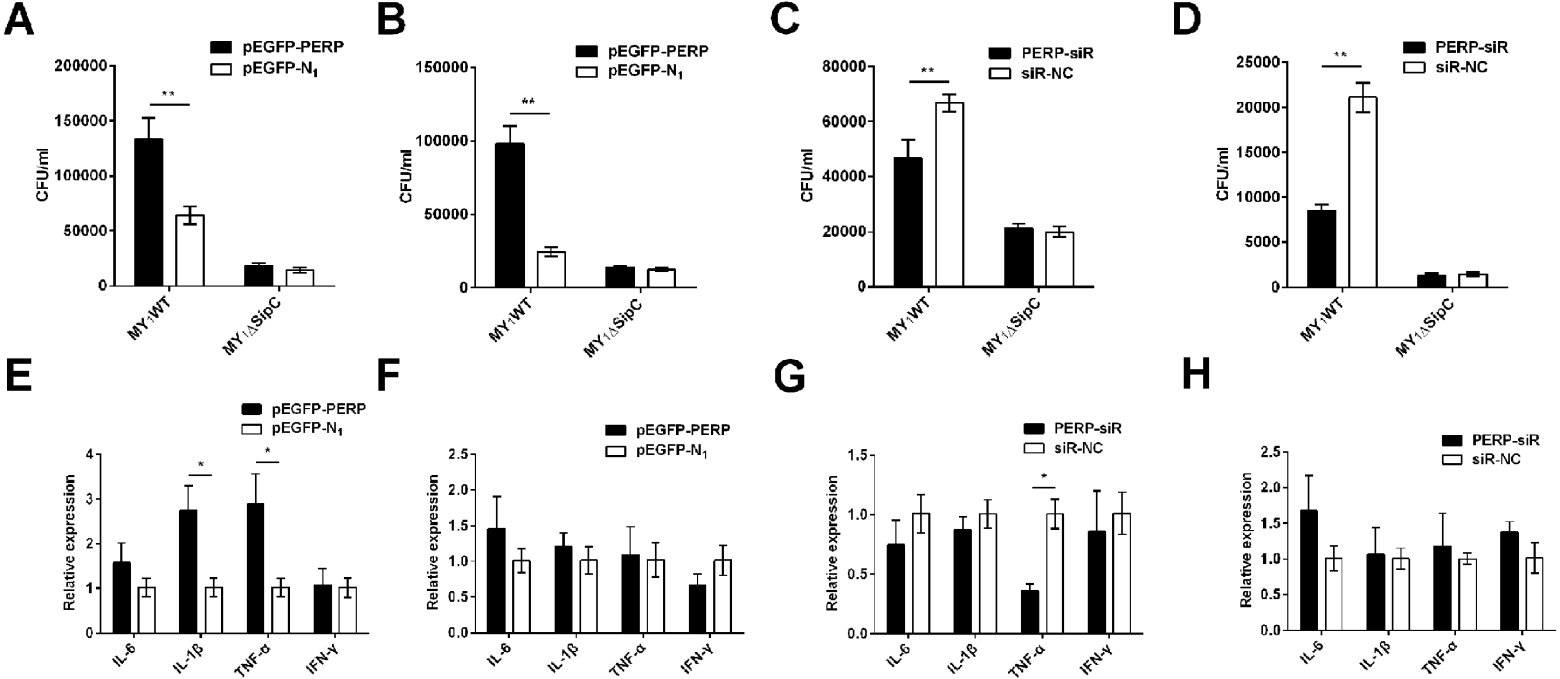
PERP interacted with SipC promotes SE adhesion and invasion to dGCs. (A) Overexpression of PERP increased SE (MY_1_WT) infection by adhesion assays. (B) Overexpression of PERP increased SE (MY_1_WT) infection by invasion assays. (C) Knockdown PERP decreased SE (MY_1_WT) infection by adhesion assays. (D) Knockdown PERP decreased SE (MY_1_WT) infection by invasion assays. (E) Overexpression of PERP enhanced the expression of duck immune response genes *IL-1β* and *TNF*-*α* during SE (MY_1_WT) infection. (F) Overexpression of PERP showed no significant change of duck immune response gene expression during SE (MY_1_Δ*sipC*) infection. (G) Knockdown PERP decreased the expression of duck immune response genes *TNF*-*α* during SE (MY_1_WT) infection. (H) Knockdown PERP showed no significant change of duck immune response genes during SE MY_1_Δ*sipC* infection compared to negative control group. Data are expressed as mean ± standard deviation of triplicate experiments. The values shown are expressed as the mean ± SD (*n* = 3). *P < 0.05; **P < 0.01. WT, wild-type.

To verify the function of PERP interacted with SipC in SE adhesion and invasion, plasmid pEGFP-PERP transfection was performed with dGCs at 90% confluence using Lipofectamine 2000. After 4 to 6 h transfection, the media was changed to serum-free DMEM. Then cells were treated with MY_1_WT and MY_1_Δ*sipC* to detect the amount of bacteria. We found a significant increase after 1 h post-infection (MOI = 10) in the number of SE MY_1_WT infecting PERP-overexpressing dGCs compared with that of control dGCs (*P* < 0.01) (Figs. 3A–3B, Table S1). Then, we applied siRNA-RNAi systems to specifically suppress PERP in dGCs. The ratio of siRNA-treated cells with green fluorescent protein expression exceeded 80%, indicating successful infection. PERP knockdown followed decreased SE adhesion and invasion by treatment with MY_1_WT in dGCs, respectively (*P* <0.01) (Figs. 3C–3D, Table S1). These results suggest that PERP may play a role in promoting the adhesion and invasion of SE MY_1_WT.

The expression of duck immune response genes *IL-6*, *IL-1β*, *TNF*-*α*, and *IFN*-*γ* was also be detected when PERP-overexpressing or knockdown in dGCs during treatment with MY_1_WT or MY_1_Δ*sipC*, respectively. There was a significant increase level in immune response of *IL-1β* and *TNF*-*α* in infecting MY_1_WT in PERP-overexpressing dGCs compared with that of control dGCs (*P* <0.05) (Fig. 3E, Table S2). When PERP knockdown followed by treatment with MY_1_WT in dGCs, there is an opposite trend emerged of the mRNA expression level of *TNF-α* (*P* <0.05) (Fig. 3G, Table S2), however, overexpression of PERP or knockdown PERP showed no significant mRNA level change of *IL-6*, *IL-1β*, *TNF*-*α*, and *IFN*-*γ* during MY_1_Δ*sipC* infection (Figs. 3F and 3H, Table S2).

### TAB2 interacts with SipC to promote SE activating inflammatory to dGCs

**Figure 4:**
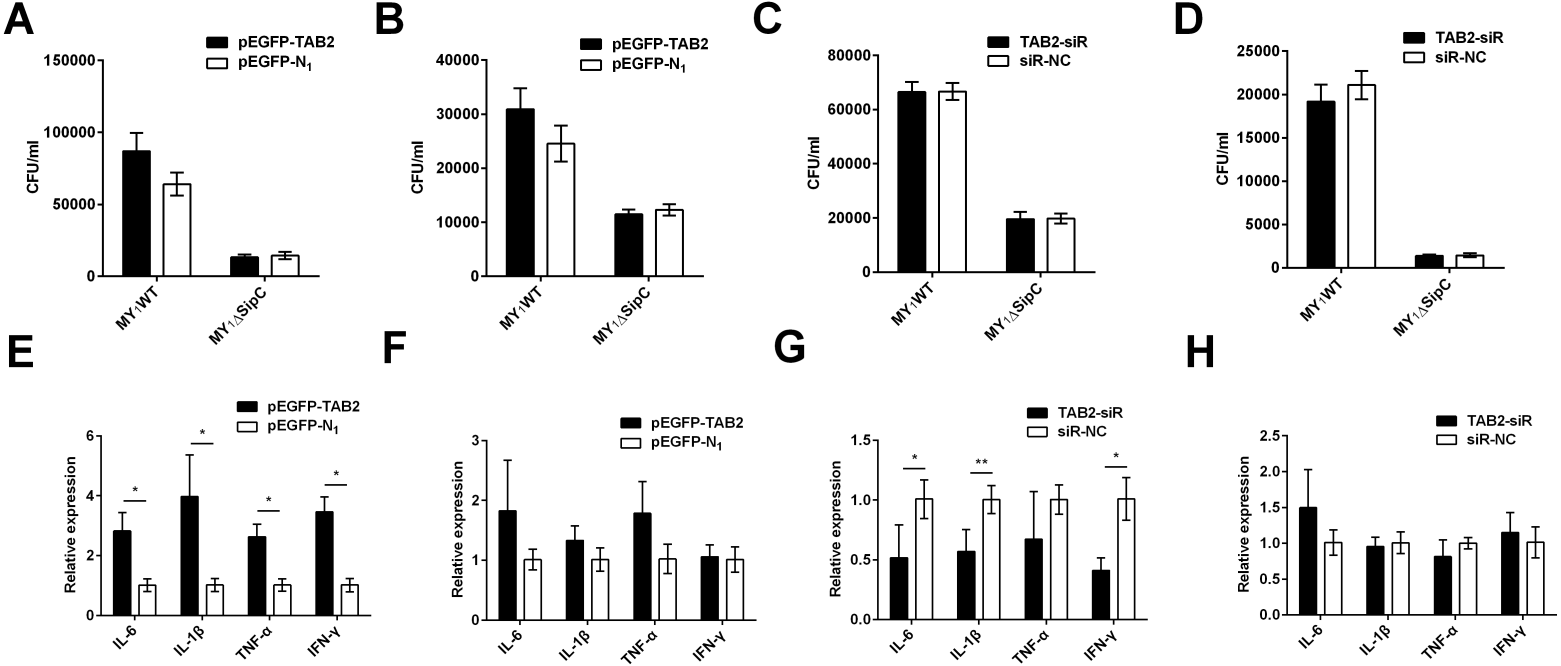
TAB2 interacted with SipC promotes SE inflammatory to dGCs. (A, C) Overexpression of TAB2 (A) or knockdown TAB2 (C) showed no significance in adhesion assays during SE (MY_1_WT or MY_1_Δ*sipC*) infection. (B, D) Overexpression of TAB2 (B) or knockdown TAB2 (D) showed no significance in invasion assays during SE (MY_1WT or MY_1_ Δ *sipC*) infection. E, G Overexpression of TAB2 (E) or knockdown TAB2 (G) influenced the expression of duck immune response genes *IL-6*, *IL-1β*, *TNF*-*α*, and *IFN*-*γ* during SE (MY_1_WT) infection. (F, H) Overexpression of TAB2 (F) or knockdown TAB2 (H) showed no significant expression of duck immune response genes *IL-6* during SE (MY_1_Δ*sipC*) infection. Data are expressed as mean ± standard deviation of triplicate experiments. The values shown are expressed as the mean ± SD (*n* = 3). *P < 0.05; **P < 0.01. WT, wild-type.

Similarly, we overexpressed and knockdown TAB2 in dGCs treated with MY_1_WT or MY_1_Δ*sipC* to determine the effects of SE adhesion and invasion and simultaneously detected the expression of duck immune response genes *IL-6*, *IL-1β*, *TNF*-*α*, and *IFN*-*γ* in MY_1_WT- or MY_1_Δ*sipC*-infected dGCs, respectively. We found that there was no significant difference after 1 h post-infection (MOI = 10) in the amount of SE MY_1_WT infecting TAB2-overexpressing dGCs compared with that of control dGCs in both adhesion and invasion process (Figs. 4A–4B, Table S1). Analogously, the number of SE in the knockdown group showed no significant difference compared with that of control group (Figs. 4C–4D, Table S1).

For the mRNA expression of some representative inflammatory gene, *IL-6*, *IL-1β*, *TNF*-*α*, and *IFN*-*γ* within TAB2-overexpressing dGCs infected by MY_1_WT were significantly higher than that of the control group (*P* <0.05) (Fig. 4E, Table S2). In contrast, when TAB2 knockdown followed by treatment with MY_1_WT in dGCs significantly decreased the expression levels of *IL-6*, *IL-1β*, and *IFN*-*γ* compared to the control group (*P* < 0.05) (Fig. 4G, Table S2). Meanwhile, MY_1_Δ*sipC* infection had no influence in the mRNA expression of inflammatory gene regardless of TAB2 knockdown or overexpression (Figs. 4F and 4H, Table S2). These results suggest that TAB2 may play a role in activating the production of inflammatory response during the SE MY_1_WT infection.

## Discussion

GCs are the layer closest to the yolk in the preovulatory follicles. SE-infected GCs may contaminate ovaries during the laying cycle, contaminating the egg and causing vertical transmission of SE. SE infection may imperil the proliferation and differentiation of GCs, causing follicular degeneration and decreased poultry egg production. Attachment of SE to a susceptible host cell surface is an essential step to establishing infection. Interestingly, attachment of SE to chicken GCs is partly inhibited by anti-chicken fibronectin (Thiagarajan et al., 1996). Studies should identify the binding site for colonization in duck ovaries during transovarian SE transmission.

To assess the binding domain between SE and dGC in the attachment process, yeast two-hybrid was used to screen for proteins mediating this interaction. In this study, we used SipC as a bait and isolated 12 co-regulatory factors that interacted with SipC from a dGC cDNA library. PERP, and other eleven *Salmonella* SipC-interacting proteins were first found involved in *Salmonella* infection. PERP was identified as a p53 effector and has since been shown to have roles in development, caspase activation, and cancer (Attardi et al., 2000; Davies et al., 2009; Ihrie et al., 2005; Paraoan et al., 2006). PERP is involved in the adhesion of SE and apoptosis of small intestinal epithelial cells in a study comparing SE-resistant and SE-sensitive chickens by microarray profiling analysis, which was also found on dGCs of laying ducks (Chausse et al., 2014; Zhang et al., 2019). In addition, PERP participates in the integrity of epithelial cells and pro-inflammatory pathways by inducing PMN migration and caspace-3 activation during *Salmonella* infection (Ihrie et al., 2005; Srikanth et al., 2010). These results suggest that PERP may play a promote role in the SE invasion of host cells.

Another novel protein TAB2, the TGF-*β* activated kinase 1-binding protein 2, has recently been implicated in IL-1 signaling (Ninomiya-Tsuji et al., 1999; Takaesu et al., 2000), and TAB2 translocated to the cytosol upon stimulation with IL-1, linking TRAF6 to TAK1 and TAB1, thereby activating TAK1 (Takaesu et al., 2000). Some investigators (Malinin et al., 1997) have suggested that activated TAK1 triggers the NF-κB-inducing kinase (NIK) and I κB kinase cascade, leading to NF-κB activation. In this study, the results also indicated that TAB2 interacted with SipC, which was characterized as adapter proteins essential for TAK1 activation in inflammatory responses. In addition, overexpressing TAB2, promoted the level of *IL-6*, *IL-1β*, *TNF*-*α* and *IFN*-*γ* mRNA during treatment with MY_1_WT, and knockdown TAB2 showed the opposite trend, however, when dGCs stimulated with SE MY_1_Δ*sipC*, the *IL-6*, *IL-1β*, *TNF-α* and *IFN*-*γ* mRNA expression showed no difference, which may be that intracellular protein TAB2 correlated tightly with SE-induced NF-κB inflammation activation. This was first report about TAB2 may interacting with SipC to co-regulate the inflammatory response during SE infection. In this study, we confirmed binding between the key host protein PERP/TAB2 and the T3SS-1 translocon component SipC by GST pull-down and *in vitro* evaluation. This observation of PERP was also reported in the HCT8 cell line, in which GST-labeled C-terminal ST SipC pulled down PERP (Hallstrom et al., 2015; Nichols & Casanova, 2010). SipC can promote the transmembrane enrichment and membrane vesicle fusion process of PERP, facilitating *Salmonella* typhimurium invasion through migration of PERP-enriched vesicles from the recycling endosome to the intestinal epithelial cell surface (Hallstrom & McCormick, 2016; Hallstrom et al., 2015). We found that PERP overexpression in dGCs enhanced SE adhesion, which was decreased after knocking out SipC *in vitro*. Thus, there was a relationship between expression of PERP and SE SipC in dGCs.

SipC is required for the translocation of *Salmonella* effectors into the host cell and for *Salmonella* invasion (Myeni & Zhou, 2010). SipC mediates exocyst formation at sites of *Salmonella* invasion via interactions with multiple components (Nichols & Casanova, 2010). The actin nucleation activity of ST SipC plays an important role in *Salmonella*-induced membrane folds and invasion. High expression of *sipC* in ST-infected BALB/c mice is closely related to persistent bacterial colonization in the liver and ileum *in vivo* (Desin et al., 2011; Gong et al., 2010). In this study, we also found that, compared to the wild-type strain, the adhesion function of the SE Δ *sipC* mutant strain decreased during SE infection of dGCs *in vitro*. We speculate that SipC has similar functions in SE and ST, as both possess T3SS, which plays a key role in bacterial invasion of non-phagocytic cells.

## Conclusions

In summary, our study screened twelve co-regulatory proteins of SipC and confirmed that SE SipC interacts with duck PERP to promote bacterial invasion and with TAB2 to facilitate inflammatory responses, which provide rationale for further investigation of the mechanisms of SE transovarian transmission. These results suggest that SE colonizes preovulatory follicles by interacting with dGCs using SipC as adhesion protein and activating inflammatory protein in this process. Further mechanistic studies are required to identify the elements required for dGC colonization by SE.

##  Supplemental Information

10.7717/peerj.7663/supp-1 Supplemental Information 1 Raw data exported from the ABI 3730xl DNA automated sequencer applied for the 20 prey plasmids were sequenced and analyzed using the BLAST tool in NCBI and preparation for the detailed investigation shown Table 2 for analysis of positive gDNA cDNA library of pGBKT7-sipC and Anas platyrhynchos dGCs cDNA library.

## Additional Information and Declarations

### Data Availability

The following information was supplied regarding data availability: The raw data is available at Figshare: Chen, Guohong (2019): Screening and identification of SipC-interacting proteins in Salmonella enteritidis using Gal4 yeast two-hybrid system in duck. figshare. Dataset. https://doi.org/10.6084/m9.figshare.7844426.v1.
